# Updated Incidence of Thyroid Cancer in the North East Region of Romania after 35 Years of Chernobyl Fallout. Is There a Link between?

**DOI:** 10.3390/diagnostics11050907

**Published:** 2021-05-19

**Authors:** Laura Teodoriu, Maria Christina Ungureanu, Letitia Leustean, Cristina Preda, Delia Ciobanu, Irena Grierosu, Mioara Matei, Roxana Iacob, Cipriana Stefanescu

**Affiliations:** 1 Endocrinology Department, “Grigore T. Popa” University of Medicine and Pharmacy Iasi, 700111 Iasi, Romania; laurateodoriu@gmail.com (L.T.); letitialeustean@yahoo.com (L.L.); cpreda1@yahoo.com (C.P.); 2 Morpho-Functional Sciences I Department “Grigore T. Popa” University of Medicine and Pharmacy Iasi, 700111 Iasi, Romania; deliaku@yahoo.com; 3 Nuclear Medicine Laboratory, “Grigore T. Popa” University of Medicine and Pharmacy Iasi, 700111 Iasi, Romania; irirai@yahoo.com (I.G.); dr.roxanaiacob@yahoo.com (R.I.); cipriana.stefanescu@yahoo.com (C.S.); 4 Preventive Medicine and Interdisciplinarity Department “Grigore T. Popa” University of Medicine and Pharmacy Iasi, 700111 Iasi, Romania; mioara.matei@umfiasi.ro

**Keywords:** thyroid cancer, Chernobyl accident, North East region of Romania

## Abstract

Thyroid cancer (TC) represents a worldwide problem, the consistent growth of the incidence increment issues about management of risk factors and curative treatment. Updated statistical data are not complete in the North East region of Romania and need to be improved. Therefore, through this study, we aim to renew the existing data on thyroid cancer. We conducted a retrospective study covering a period of 10 years. Data were collected from a hospital information system (InfoWorld) between 2009 and 2019. Patients’ age groups were stratified in relation with the age at the moment of the Chernobyl event. A database was obtained (Microsoft Excel) and statistical correlations were applied. In the studied period, 1159 patients were diagnosed: 968 females and 191 males, distributed by region, with the highest addressability in Iasi (529), followed by neighboring counties. Age distribution displayed that most of the thyroid cancers were in the range 4060 years old (50.94%), followed by 60–80 years old (32.41%). Most patients were diagnosed with papillary carcinoma 63.10%, then follicular 14.7%, medullary 6.74% and undifferentiated 1.02%. Romania was in the vicinity of the radioactive cloud at Chernobyl fallout, so we must deliberate whether the increased incidence of thyroid cancer in the age group 40–60 years is associated with radiogenicity (iodine 131) given the fact that over has 35 years and the half-life of other radioisotopes like Caesium-137 and Strontium -90 is completed.

## 1. Introduction

**Figure 1:**
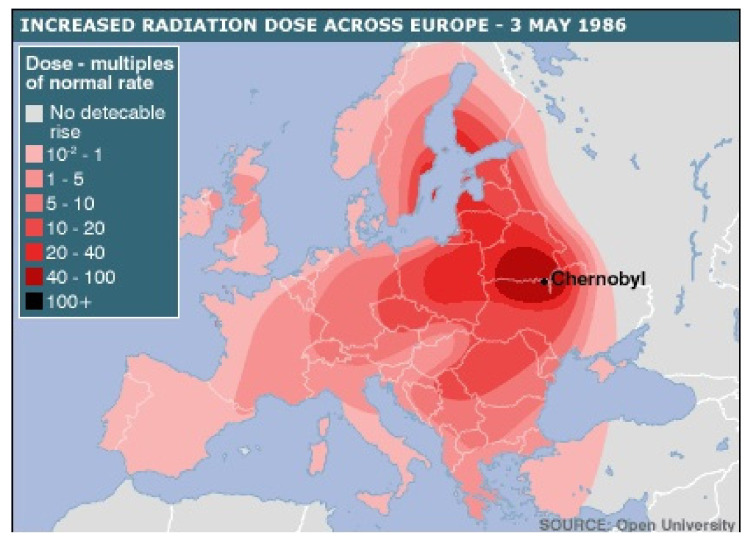
Chernobyl fallout radiation cloud on 3 May 1986 covering a map of Europe [9].

The incidence of thyroid carcinoma is increasing worldwide (3% of all cancer incidence) [1] and well differentiated thyroid carcinoma is expected to be the fourth most common cancer in 2030 [2]. Age-standardized incidence rate (ASIR) showed an upward trend worldwide, as written by Deng et al. The three highest incidence countries are China: 11,016 patients in 1990 and 41,511 in 2017, United States: 10,833 patients in 1990 and 25,896 in 2017 and India: 7369 patients in 1990 and 25,675 in 2017. An increased incidence was seen in 34% cases of high socio-demographic index areas [3], inversely proportional to the socio-demographic index being the cases of relative mortality in thyroid cancer [4]. Increased incidence has also been reported in Europe with an overall 5-year age-standardized relative survival of 88% in women and 81% in men. In the latter case, 5-year relative survival was 5–7 percentage points lower than in women in most European countries [5]. Age-standardized deaths rates (ASDR) studied all over Europe showed that, in our country, results for ASDR were similar between the years 2000 (0.33 for men and 0.43 for women) and 2010 (0.3 for men and 0.43 for women), meaning that deaths caused by thyroid cancer were stationary over time [6]. In addition to inherited molecular changes for risk factors associated with increased incidence of thyroid cancers, it also includes molecular changes caused by radiation [7,8]. The biggest nuclear accident in the world was the Chernobyl fallout in 1986, with the radioactive cloud traveling 250,000 km^2^ to northwestern Europe. Romania is located in the immediate vicinity of Chernobyl, more precisely the northeastern part of Romania where we conducted this study (Figure 1) [9]. 

Considering that current information, after the Chernobyl fallout, showed that the incidence of thyroid carcinoma is increased in those exposed to nuclear radiation [7], our aim is to show whether the incidence of thyroid cancers in the North East region of Romania in the past 10 years is related to Chernobyl radiation exposure or not. The main reason for this information is to complete the worldwide map with updated results.

## 2. Materials and Methods

We conducted a retrospective study between the years 2009 and 2019. Data were collected from “St. Spiridon” University Hospital information system (InfoWorld) by key words such as thyroid carcinoma that represented admission diagnosis for our patients. In accordance with WHO standards, pathologies were coded according to the 3rd edition of the International Classification of Diseases for Oncology (ICD-O-3). For our analyses, we used primary invasive thyroid cancer cases (ICD-O-3: C73) recorded between the years 2009 and 2019. Thyroid cancers were grouped by histological subtype papillary: ICD-O-3 codes M8050/0, M8050/2, M8050/3, M8050/6, M8260, M8341, M8342, M8344, M8350; non-papillary: ICD-O-3 codes M8020, M8021, M8030, M8031, M8032,M8033, M8041, M8290, M8330, M8331, M8332, M8335, M8345, M8346, M8347, M8510, M8511 and M8512; rare (<1%) and unknown types (others/unknown) and tumor stage (early, advanced) according to recent oncology guidelines, using the TNM classification according to American Joint Committee on Cancer (AJCC) Cancer Staging Manual (7th edition) [10]. In people younger than 45 years of age, stage I (M0) tumors were defined as early cancer and stage II (M1) tumors were defined as advanced cancer. In people aged 45 or older, stage I (T1N0M0) and stage II tumors (T2N0M0) were defined as early cancer, whereas stage III (T3N0M0, T1–3N1aM0) and stage IV (M1, all anaplastic carcinoma) tumors were defined as advanced cancer [11].

Inclusion criteria contained all malignant thyroid histologies and the exclusion criteria were benign thyroid pathologies.

The inventory list of this research was obtained in print format following ICD-O-3 codes, after which we selected and eliminated the duplicate admissions from the monitoring program, leaving only the initial hospitalization. When the database was complete with initial hospitalization dates and the histopathological results of our patients, we used descriptive methods to analyze the incidence and statistical functions were applied to achieve the following results.

Statistical analysis: Rates were age-standardized to the Romanian standard population as published by the National Institute of Statistics www.insse.ro. We figured annual thyroid cancer incidence rates stratified by sex, histological subtype and tumor stage. We fitted a linear regression model to estimate the annual mean absolute and relative changes in the standardized rates, with calendar year as predictor variable. A Pearson’s chi-squared (χ2) test was performed to determine the differences in age stratification and histopathological factors between the groups. Continuous outcomes were analyzed using independent t-tests for groups of two and one-way analysis of variance among groups of three of more. *p* < 0.05 was considered significant. Statistical analyses were performed with Excel.

Ethics: Anonymized and publicly available aggregated data were used for our analyses. There was no threat to patient confidentiality. According to the Swiss Human Research Act (Humanfoschungsgesetz HFG), no ethical approval or trial registration is needed for such analyses.

## 3. Results

**Figure 2:**
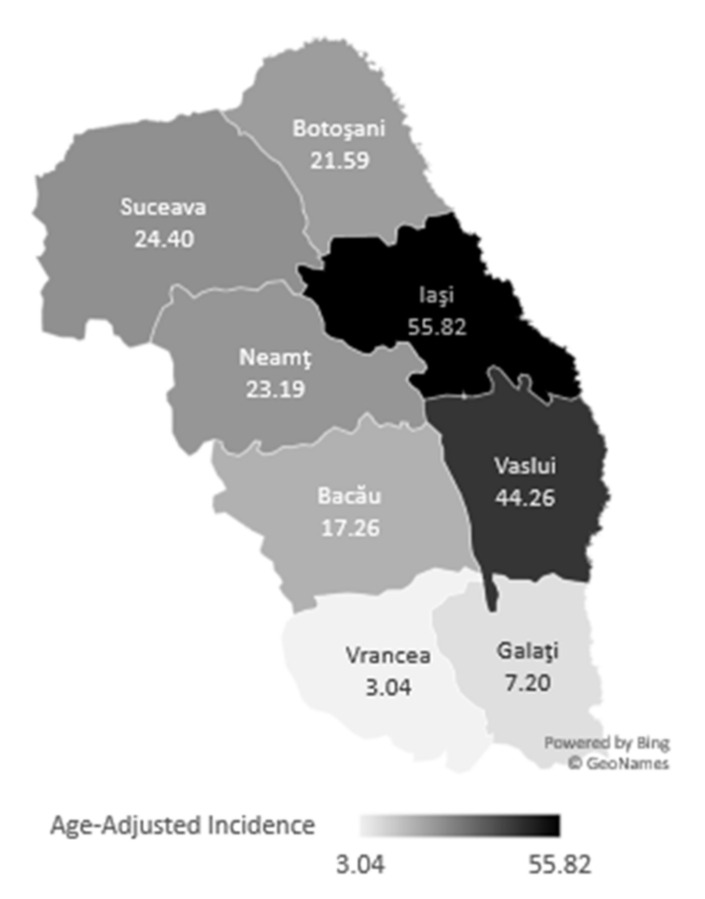
Age-adjusted thyroid carcinoma incidence in North East counties of Romania [12].

We included 1159 patients following key words: thyroid carcinoma search (C73). Of them, 191 (16.48%) were men and 968 (83.52%) women. Stratifications by counties highlights that a majority of 529 (45.64%) were from Iasi, where the Endocrinology University center of the North East region in Romania is located, followed by vicinity counties. Age-adjusted incidence of thyroid carcinoma displayed increased age-adjusted results for Iasi county, followed by vicinity county Vaslui. These two counties have the most patients diagnosed with thyroid carcinoma per 100,000 inhabitants (Figure 2).

Age-adjusted incidence was obtained using public statistical data obtained from the Romanian National Institute of Statistics using interrogations on Tempo Online Platform [12]. 

Histopathological results (1068 results found) showed that papillary carcinoma was the most common with a total of 63.10% (classic variant of papillary carcinoma and multiple endocrine neoplasia type 1), followed by follicular carcinoma (14.7%), follicular variant of papillary thyroid carcinoma (10.67%), medullary carcinoma (6.74%) (sporadic medullary carcinoma and multiple endocrine neoplasia type 2), Hürthle cell carcinoma (2.43%), anaplastic thyroid carcinoma (1.02%), thyroid primary lymphoma (0.56%) and poor differentiated thyroid carcinoma (PDTC) (0.28%). For syndrome association of thyroid carcinoma, multiple endocrine neoplasia 1 (MEN) had papillary carcinoma in 0.18% and MEN 2 had medullary carcinoma in 0.28% (Table 1).

**Figure 3:**
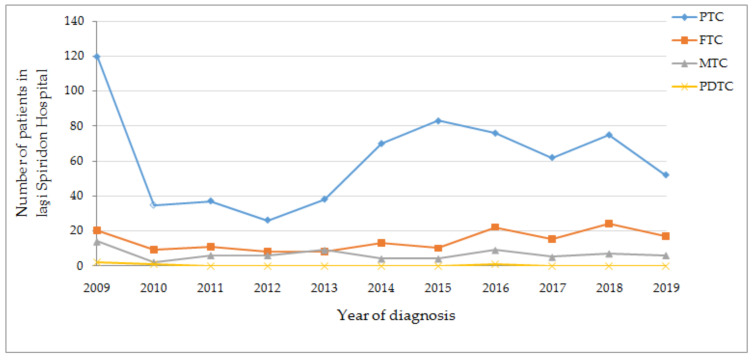
Thyroid carcinoma’s histopathological picture of the North East region (2009–2019), PTC—papillary thyroid carcinoma, FTC—follicular thyroid carcinoma, MTC—medullary thyroid carcinoma, PDTC—poorly differentiated thyroid carcinoma.

Highest annual incidence was found for papillary carcinoma in 2009 (120), right at the beginning of the study; after that, incidence suffered some peaks over the years, as shown in Figure 3. Remaining histological subtypes were in plateau.

We found that over the studied period, patients were diagnosed with thyroid carcinoma at the age of 50–60 years old, with a stable male to female ratio over the time (Table 2).

Mean age at diagnosis over each year of study results was approximately 50 years old. Considering that the peak of thyroid incidence was in 2009, mean age in that year was 51.7 for female and 53.9 for males. Those patients were about 28.7 years old for female and 30.9 years old for males at the time of the Chernobyl accident. We found 48 cases in 2009 (year with a peak in incidence) who were children and young adults at the time of the nuclear fallout (0–20 years old) (Table 2).

Patients with well differentiated thyroid carcinomas, 971 patients (90.91%), received radioactive iodine treatment in approximately 24.71% cases. TNM staging results showed 504 evaluated results, of which the most common was pT1 (34.92%), followed by pT3 (18.45%), pT2 (4.16%) and pT4 (0.39%). Lymph node involvement was found as N1 in 12.3% cases and N2 in 0.39% cases. Secondary lesions were encountered in 0.39% patients with valid histopathological results.

To investigate the influence of patients’ age on histological subtype of cancer, we divided all patients by age groups. This classification displayed that per both sexes, papillary thyroid carcinoma was the most encountered. The peak of thyroid carcinoma was detected in the age group of 51–60 years old (296 patients): 40 males and 256 females. Of them, papillary carcinoma was found in 75% for both sexes (Table 3).

According to TNM staging with a cutoff value of 45 years old, there were significant statistical results for papillary thyroid carcinoma between groups under 45 and over 45 years old. There were no differences in the distributions of gender, lesions and lymph node metastasis (Table 4).

However, regarding the distribution of histological types, the extended age group classification table shows that significant statistical results were found for papillary subtype with lymph node invasion and distant metastasis (Table 5). Lymph node invasion was due to medullary carcinoma in nine patients, whilst rest of the cases suffered papillary lymph node metastasis.

## 4. Discussion

Our results are in accordance with previous studies that have shown close outcomes regarding histological findings [5]. As in EUROCARE-5, we identified that the most common thyroid cancer is papillary carcinoma, followed by follicular type, medullary type and undifferentiated carcinomas. Similar results showed that 63.10% were papillary carcinomas in our study compared to a total of 71% in EUROCARE-5; also, 14.7% were follicular carcinomas compared to a total of 15% in the European study, displaying almost identical result between these two types of carcinomas. Medullary carcinoma was encountered in 6.74% of cases in our study, more cases compared to the total of 5% in the European study. Comparing with Eastern Europe’s results, our numbers showed that papillary carcinoma incidence was similar between our region and Estonia and Latvia, and follicular carcinoma was similar between our region and Bulgaria, Czech Rep., Latvia, Estonia and Slovakia. Medullary carcinoma was similar to that of Estonia, Latvia and Poland. Anaplastic carcinoma was identified in 1.02% of those in our study, fewer cases than EUROCARE-5, which encountered a total of 3%, and a similar result with the 2% encountered in Lithuania. We were able to include more histological subtypes such as: follicular variant of papillary carcinoma, Hürthle cell carcinomas, poorly differentiated carcinomas and MEN, because of relatively small group of patients in this study compared to the large groups included in EUROCARE-5 [5]. We also classified our histological findings by TNM staging, and the most common stage that we found was pT1 34.92%, fewer cases than those encountered by Lim et al. (67.4%), those results being explained by the huge difference in patient numbers included in each study [13]. Comparative results, considering the patient number gap, were obtained for pT2 (4.16% in our study and 7.6% in the other study) and pT3 (18.45% in our study compared to 12%). We observed similar results to Lim et al. regarding sequence of TNM percent: pT1 and then pT3 [13].

Sex incidence is also in accordance with the literature. It is no surprise that our study identified 83.52% women and 16.48% men. These results are similar to those encountered by our colleagues from Cluj in Romania: 88.8% females and 11.2% males [14]. Our female incidence is slightly higher than the worlwide incidence (77.1%) and male incidence is lower in our region than the worldwide incidence (29.9%) [4].

Age-adjusted incidence shown that Iasi and Vaslui counties had the most patients per 100.000 inhabitants diagosed with thyroid carcinoma. In Iasi is located the only University Endocrinology Clinic in the North East region of Romania, so most of the patients were adressed here, especially those from vicinities such as Vaslui. Other close counties had a lower age-adjusted incidence because they might refer to other Endocrinology centers (Cluj, Bucharest) due to more fluid infrastructure. Furthermore, for Botosani county, infrastructure is problematic; therefore, patients lack proper medical surveillance.

These results complete our collegues work that highlights the same events reported between 1970 and 1985 before the Chernobyl fallout and after exposure during 1986–1992. Before the Chernobyl accident they encountered a total of 94 thyroid carcinomas: 33.84% papillary, 34.78% follicular, 7.52% medullary and 12.22% anaplastic. After Chernobyl exposure (1986–1992) numbers changed, as they encountered 101 thyroid carcinomas: 47.47% papillary, 16.16% follicular, 4.04% medullary and 34.34% anaplastic. Major change consists in increased undiferentiated histology after the Chernobyl fallout, and over the years this histology wasn’t connected to radioactive the accident, with the main reason being increased adresability after this important event [15].

Another study conducted in our endocrinology department showed that between 2001 and 2004 125 patients were diagnosed with papillary carcinoma and after that, between 2005 and 2008, 276 patients were diagnosed with papillary carcinoma [16]. Unfortunately, we were confronted with a lack of information regarding thyroid carcinoma’s incidence between the years 1993 and 2000.

**Figure 4:**
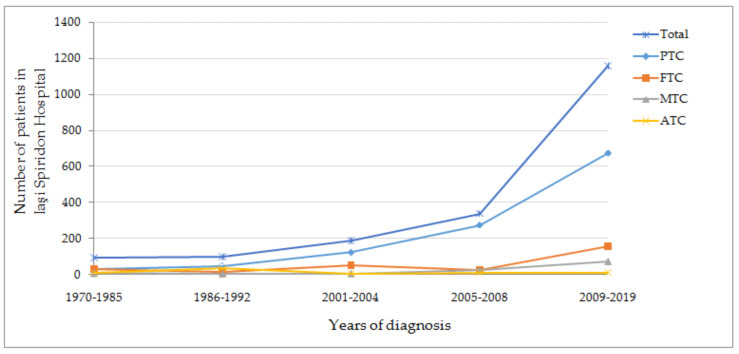
Thyroid carcinoma’s evolutionary timeline before Cernobyl fallout and after nuclear exposure. Combined results of Mogos et al. (1970–1992), Buzduga et al. (2001–2008) and our results. The period of 1993–2000 is missing from this figure [15,16]; PTC—papillary thyroid carcinoma, FTC—Follicular thyroid carcinoma, MTC—medullary thyroid carcinoma, ATC—anaplastic thyroid carcinoma.

Combined results of these three studies, conducted in the same endocrinology department, show that papillary carcinoma has considerably increased incidence over time, followed by follicular carcinoma in a smaller proportion, as seen also in Miranda-Filho et al. [1]. Medullary and anaplastic carcinomas remained in plateau in our analysis (Figure 4).

Age stratification highlights that 50.94% were between 40 and59 years old, followed by 32.41% of those 60–80 years old. Those many cases in the first category were children and young adults (6–25 yo) at the time of the Chernobyl fallout. Mean age at diagnosis in 2009, the year with most frequent cases, was 50 years old, meaning that at the time of exposure they were 30 years old.

Our result also highlight that about 312 (26.91%) of total patients were between 0 and 20 years old at the time of the Chernobyl accident. Radiation exposure in childhood involves a great risk in developing thyroid carcinoma and studies have shown that children and young adults were most affected after this nuclear accident [17].

All Romanian studies of thyroid carcinoma incidence were reviewed, and the results showed that the literature reports an increased incidence of 2–5 times depending on each author. Maximum incidence of radiogenic thyroid carcinoma appeared within 5–10 years of the disaster, with a larger potential of 1–20 years, depending on the exposure rate. Our data completes these results showing a constant increase in thyroid carcinoma over 30 years after the Chernobyl fallout [18]. Piciu et al. show a vast increase of thyroid carcinoma cases in Romania since 1970, with a maximum increase of 511% between the years 2001 and 2010 in contrast to 1970–1980. Our maximum incidence was in 2009, 23 years after this nuclear accident [14].

Patients exposed to fallout radioisotopes were monitored over time, and comparative studies between radiogenic thyroid carcinomas and sporadic ones arise. Many studies came from Ukraine, and Bogdanova et al. monitored patients that were <4 years old at the moment of the accident, living in the exposed area, and found that thyroid carcinomas were more aggressive than sporadic ones with a trabecular solid pattern. The comparison was made with patients born after the Chernobyl accident. Fusion oncogene drivers may confer higher tumor aggressiveness than point mutation in young patients, and that may be the response to radiogenic aggressiveness [19].

Further studies showed that radioactive iodine exposure in utero may affect children in a manner of dose dependent Patients exposed in utero were monitored and some of them developed nodular goiter with significant results ofnodule dimensions increased by >1 cm. Some of them had thyroid carcinoma, but these results were not statisticaly significant. These publications highlight that we should include as a risk factor ^131^I in utero exposure for nodular goiter [20].

Thirty years after Chernobyl, nearly 11,000 thyroid cancer cases have been reported among those who were children or adolescents at the time of the accident in Belarus, Ukraine and the most contaminated regions of Russia [17].

There has unquestionably been a substantial dose-dependent excess risk of Iodine-131 related radiogenic thyroid cancer, many with a distinctive histopathology RET/PTC3. RET fusions are reported to be the most common observed alteration in children thyroid carcinoma, and appeared to occur in approximately 25–30% of sporadic pediatric PTC; these results further increase to nearly 45% in patients exposed to radiation [21].

Results of thyroid cancer incidence following exposure in utero have suggested that the risk is the same, or larger, than that following similar exposures in infancy. Investigation of clean-up workers has produced new data on thyroid cancer risk in exposed adults, suggesting that the risk observed in exposed adults is not much smaller than those observed in children [22].

Also, a retrospective study was conducted in Romania that investigated the potential in utero risk factor for thyroid carcinoma in the pediatric population. Children included in this study were born after 1986. The study found that thyroid carcinoma had a peak at ages of 10–13 years old after the nuclear accident [23].

Follow-up after Chernobyl exposure was done in the affected areas, and over a period of 23 years (1989–2012), age-standardized thyroid carcinoma incidence in females increased by 3.7 times in the high exposure region, and by 2.9 times in the low exposure region. Males also suffered an increase by 3.5 times in high areas, and 1.82 times in low areas. Substantial differences were observed in the age group 40–49 years old. These results are similar to our findings concluding that amongst patients who were children at the moment of the exposure a high dose radiation may trigger over time a thyroid carcinoma [24].

In Romania, few studies covered the Chernobyl radiogenic thyroid carcinoma subject, but an important follow-up was made in Targu Mures, an area that was affected by radiation. They found that between 1984 and 2007, amongst patients divided by pre-Chernobyl accident and post-Chernobyl accident, papillary carcinoma increased over time. After a period of 5–8 years from the radiogenic event, they marked first 10 children diagnosed with papillary carcinoma, noting that before the accident there were no children diagnosed. The incidence of thyroid cancer increased 3–5 times in 1993–1998 compared to data registered before exposure to ionizing radiation from Chernobyl. Our findings supplement these results showing that papillary incidence is still increased over time, and most diagnosed patients were children and young adults at the time of the exposure [25].

Caesium 137 was measured in our country by specialists using γ spectrometric measurements on soil samples collected from 153 locations. Results show that Caesium had an average of 8.3 ± 0.2 kBqm^−2^, with more elevated values in the mountain areas (18.3 ± 0.6 kBqm^−2^) compared to the hills and plains (2.6 ± 0.1 kBqm^−2^). This conclusion covered only one region of Romania, and the North East region was not studied at the time [26].

**Figure 5:**
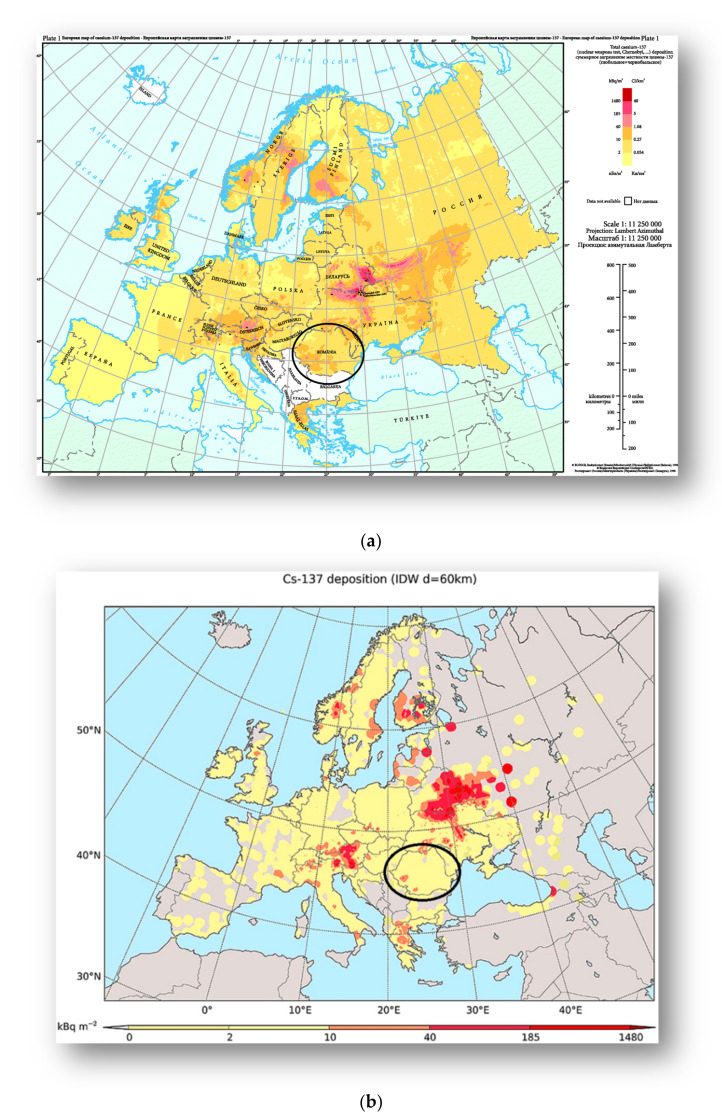
(**a**) Caesium 137 deposition from the Atlas of Caesium Deposition on Europe after the Chernobyl Accident EUR report nr. 16733, De Cort et al. 1998 updated in 2012 by European Environment Agency (EEA) [27]; (**b**) Updated map (2016) of Cs–137 deposition in Europe, provided from Evangeliou et al. based on 11,334 measurements in 2016, adapted with permission from [28]. Copyright 2016, Elsevier.

Other result of Caesium 137 exposure over Europe’s map show that Romania has an exposed background of 2–10 kBq m^−2^ and some peaks of 10–40 kBq m^−2^ in our region of interest: the center and north location, as seen in Figure 5 [27,28].

A timeline study focused on the 2009–2019 period, which means that the Chernobyl event occurred 23–33 years before, comes in line with the half-life of the other radioisotopes such as Caesium-137 and Strontium-90, not specifically involved in thyroid carcinoma, but may be involved in other carcinogenesis. This study is the beginning of our research in the field of synchronous and metachronous cancers linked to radiogenic events.

## 5. Conclusions

Our work results bring many new insights on thyroid carcinoma incidence, and details on patients’ age and potential exposure to the Chernobyl fallout fill many gaps in our country’s cancer registry. We should develop a proper follow-up system for patients born in and after 1986, both in utero patients and children at the time of exposure. It will be interesting to investigate their lifestyle and other risk factors to conclude if thyroid cancer was linked specifically to the Chernobyl nuclear accident or not. Molecular testing, such as RET/PTC3, might be helpful and doable in further studies.

## Figures and Tables

**Table 1:** Incidence of histopathological characteristics in 1068 patients.

Histological Characteristics	Number of Patients (%)
Papillary carcinoma:	
Classical variant	672(62.92)
MEN 1 associated	* 2 (0.18)
Medullary carcinoma:	
Sporadic	69 (6.46)
MEN 2 associated	* 3 (0.28)
Follicular carcinoma:	
Classical variant	157 (14.7)
Follicular variant of papillary carcinoma	114 (10.67)
Hürthle cell carcinoma	26 (2.43)
Anaplastic carcinoma	11(1.02)
Poor differentiated carcinoma	3 (0.28)
Primary thyroid lymphoma	6 (0.56)

* Considering that final genetic diagnosis is missing for MEN, those patients will be counted in papillary and medullary sections.

**Table 2:** Timeline characteristics of this study (age, sex, histopathological).

	2009	2010	2011	2012	2013	2014	2015	2016	2017	2018	2019	*p*-Value
Aget at diagnosis												
Overall	52.0	52.1	55.5	53.7	54.6	53.3	55.9	55.8	57.0	55.1	57.4	1.000
Male	53.9	57.6	58.8	57.5	54.6	53.7	57.3	55.5	51.3	55.3	56.2	1.000
Female	51.7	50.9	54.6	52.6	54.7	53.2	55.5	55.9	57.9	55.0	57.6	1.000
*p*-value	0.911	0.590	0.773	0.717	0.909	0.956	0.949	0.886	0.462	0.939	0.812	
Sex												
Male	27	14	17	18	19	17	22	23	14	11	9	0.528
Female	177	64	68	62	74	89	89	102	89	94	60	0.998
Male to Female ratio	0.15	0.22	0.25	0.29	0.26	0.19	0.25	0.23	0.16	0.12	0.15	
Total	204	78	85	80	93	106	111	125	103	105	69	
0–20 years old in 1986 (26.91%)	48	16	23	18	16	33	29	39	27	39	24	
Pathological classification												
Papillary	120	35	37	26	38	70	83	76	62	75	52	0.259
Follicular	20	9	11	8	8	13	10	22	15	24	17	
Medullary	14	2	6	6	9	4	4	9	5	7	6	
PDTC*	2	1	0	0	0	0	0	1	0	0	0	

* PDTC—poorly differentiated thyroid carcinoma.

**Table 3:** Histopathological incidence by sex and age groups.

	<10		11–20		21–30		31–40		41–50		51–60		61–70		71–80		≥81	
Both sexes	*n*	%	*n*	%	*n*	%	*n*	%	*n*	%	*n*	%	*n*	%	*n*	%	*n*	%
Papillary	0	0	6	85.7	16	72.7	71	75.5	135	75.4	222	75	162	71.4	51	67.1	11	84.6
Follicular	0	0	0	0	2	9.1	15	16	31	17.3	48	16.2	46	20.3	13	17.1	2	15.4
Medullary	0	0	1	14.3	4	18.2	8	8.5	12	6.7	22	7.4	16	7	9	11.8	0	0.0
Anaplastic	0	0	0	0	0	0	0	0	0	0	2	0.7	3	1.3	2	2.6	0	0.0
PDTC*	0	0	0	0	0	0	0	0	1	0.6	2	0.7	0	0	1	1.3	0	0.0
Total	0	0	7	100	22	100	94	100	179	100	296	100	227	100	76	100	13	100
Males																		
Papillary	0	0	0	0.0	3	60.0	6	85.7	18	78.3	27	67.5	25	67.6	11	50.0	0	0
Follicular	0	0	0	0.0	0	0.0	0	0.0	3	13.0	8	20.0	10	27.0	5	22.7	0	0
Medullary	0	0	1	100.0	2	40.0	1	14.3	2	8.7	3	7.5	2	5.4	5	22.7	0	0
Anaplastic	0	0	0	0.0	0	0.0	0	0.0	0	0.0	2	5.0	0	0.0	1	4.6	0	0
PDTC*	0	0	0	0.0	0	0.0	0	0.0	0	0.0	0	0.0	0	0.0	0	0.0	0	0
Total	0	0	1	100	5	100	7	100	23	100	40	100	37	100	22	100.0	0	0
Females																		
Papillary	0	0	6	100.0	13	76.5	65	74.7	117	75.0	195	76.2	137	72.1	40	74.1	11	84.6
Follicular	0	0	0	0.0	2	11.8	15	17.2	28	18.0	40	15.6	36	19.0	8	14.8	2	15.4
Medullary	0	0	0	0.0	2	11.8	7	8.1	10	6.4	19	7.4	14	7.4	4	7.4	0	0.0
Anaplastic	0	0	0	0.0	0	0.0	0	0.0	0	0.0	0	0.0	3	1.6	1	1.9	0	0.0
PDTC*	0	0	0	0.0	0	0.0	0	0.0	1	0.6	2	0.8	0	0.0	1	1.9	0	0.0
Total	0	0	6	100.0	17	100.0	87	100.0	156	100.0	256	100.0	190	100.0	54	100.0	13	100.0

* PDTC—poorly differentiated thyroid carcinoma.

**Table 4:** Characteristics of thyroid cancer patients aged ≤ 45 and > 45 years as in TNM staging by 7th AJCC edition.

	<45	≥45	Total	χ^2^/F	*p*-Value
Gender					
Male	47	144	191	0.318	0.573
Female	220	748	968		
Histological Type					
Papillary	153	521	674	104.903	<0.001
Follicular	27	130	157		
Medullary	20	52	72		
Anaplastic	0	7	7		
PDTC	0	4	4		
Others	92	255	347		
LN metastasis					
Yes	18	44	62	1.434	0.231
No	44	159	203		
Metastasis					
Yes	0	2	2	0.600	0.439
No	1	3	4		

PDTC—poorly differentiated thyroid carcinoma, LN—lymph node.

**Table 5:** Characteristics of thyroid cancer patients by age groups related to the Chernobyl fallout.

	<21	21–45	46–59	≥60	Total	χ2/F	*p*-Value
Gender							
Male	3	46	56	86	191	8015	0.046
Female	9	227	380	352	968		
Histological Type							
Papillary	6	161	253	254	674	110,375	<0.001
Follicular	0	30	58	69	157		
Medullary	1	20	23	28	72		
Anaplastic	0	0	1	6	7		
PDTC	0	0	2	2	4		
Others	5	89	133	119	346		
LN metastasis							
Yes	1	19	22	20	62	55.880	<0.001
No	1	46	74	82	203		
Metastasis							
Yes	0	0	1	2	3	254.545	<0.001
No	0	4	0	0	4		

PDTC—poorly differentiated thyroid carcinoma, LN—lymph node.
